# Computed tomography for diagnosis of tiny acute myocardial infarction with microvascular obstruction

**DOI:** 10.1007/s10554-025-03325-w

**Published:** 2025-01-18

**Authors:** Kouhei Takayama, Hiroyuki Takaoka, Tomonori Kanaeda, Takashi Hiraga, Tatsuro Yamazaki, Yoshitada Noguchi, Yusei Nishikawa, Shuhei Aoki, Moe Matsumoto, Satomi Yashima, Katsuya Suzuki, Makiko Kinoshita, Kazuki Yoshida, Haruka Sasaki, Noriko Suzuki-Eguchi, Yoshio Kobayashi

**Affiliations:** 1 Department of Cardiology, Eastern Chiba Medical Center, Togane, Japan; 2 Department of Cardiovascular Medicine, Chiba University Graduate School of Medicine, Chiba, Japan

**Keywords:** Microvascular obstruction, Computed tomography, Late enhancement

A 72-year-old male was admitted to hospital complaining of back pain. He previously underwent plasty for mitral valve stenosis and persistent atrial fibrillation. He became aware of sudden onset chest and back pain that lasted for about 30 min in the evening on the day before the admission. He continued to experience slight back pain and visited our outpatient clinic the next morning. His electrocardiogram revealed no abnormal ST changes, but his serum troponin T was elevated to 0.928ng/mL (≤ 0.1 ng/mL); therefore, acute myocardial infarction (AMI) was suspected. We also suspected the embolus in a small branch of the left circumflex artery on his emergent invasive coronary angiography (ICA) (Figure A), and anticoagulant therapy was initiated. Additionally, the screening of intracardiac thrombus was considered necessary. Still, as the troponin remained elevated on initial blood sampling, transesophageal echocardiography, which is taxing on the case, and magnetic resonance imaging (MRI), which requires prolonged rest in an enclosed space, were deemed difficult, so a contrast-enhanced computed tomography (CT) was performed. Ninety-six mL iodine contrast was injected and the early phase was scanned at almost 30 s after the start of injection. CT images of the early contrast phase showed that no significant coronary artery stenosis was suspected (Figure B), but it was difficult to determine whether the thrombus in the left atrium was a contrast delay (Figure C). Therefore, the late enhancement imaging was added six minutes after the injection of contrast media and confirmed as a thrombus (Figure D). Additionally, late enhancement with microvascular obstruction (MVO) on the mid-lateral left ventricular myocardium (LVM) was detected (Figures E and F). Seven days after the onset, cardiac MRI was performed because of the evaluation of myocardial damage and the follow-up of intra-cardiac thrombus. It showed a late gadolinium enhancement with MVO in the mid-lateral LVM (Figures G and H), and a high-intensity signal of the LVM at the same point was also detected in the T2 weighted image (Figure I). We diagnosed him with AMI caused by embolization of LAA thrombus.

MVO is detected on MRI in cases with AMI, and it is known as a maker of worse prognosis [1]. MVO is a non-enhanced region most probably due to simultaneous damage and necrosis of both myocytes and capillaries in the center of the infarct-enhanced region [1]. It is easy to detect MVO on MRI. However, it is not easy to perform an MRI on patients suspected of having AMI because of the long acquisition time in the limited space, and the diagnostic accuracy of coronary artery stenosis is not enough. On the other hand, CT can be used to examine suspected cases of AMI in a short time and has high diagnostic accuracy for coronary artery stenosis, so the detection of LV delayed contrast and MVO with the addition of late-phase imaging would be very beneficial in daily clinical practice. However, late enhancement and MVO evaluation may be difficult in cases with high body mass index cases requiring contrast reduction due to renal impairment, as CT image quality is degraded [2].

This is the first report on the utility of CT for the challenging diagnosis of tiny MVO of AMI by embolization of LAA thrombus.

**Figure:**
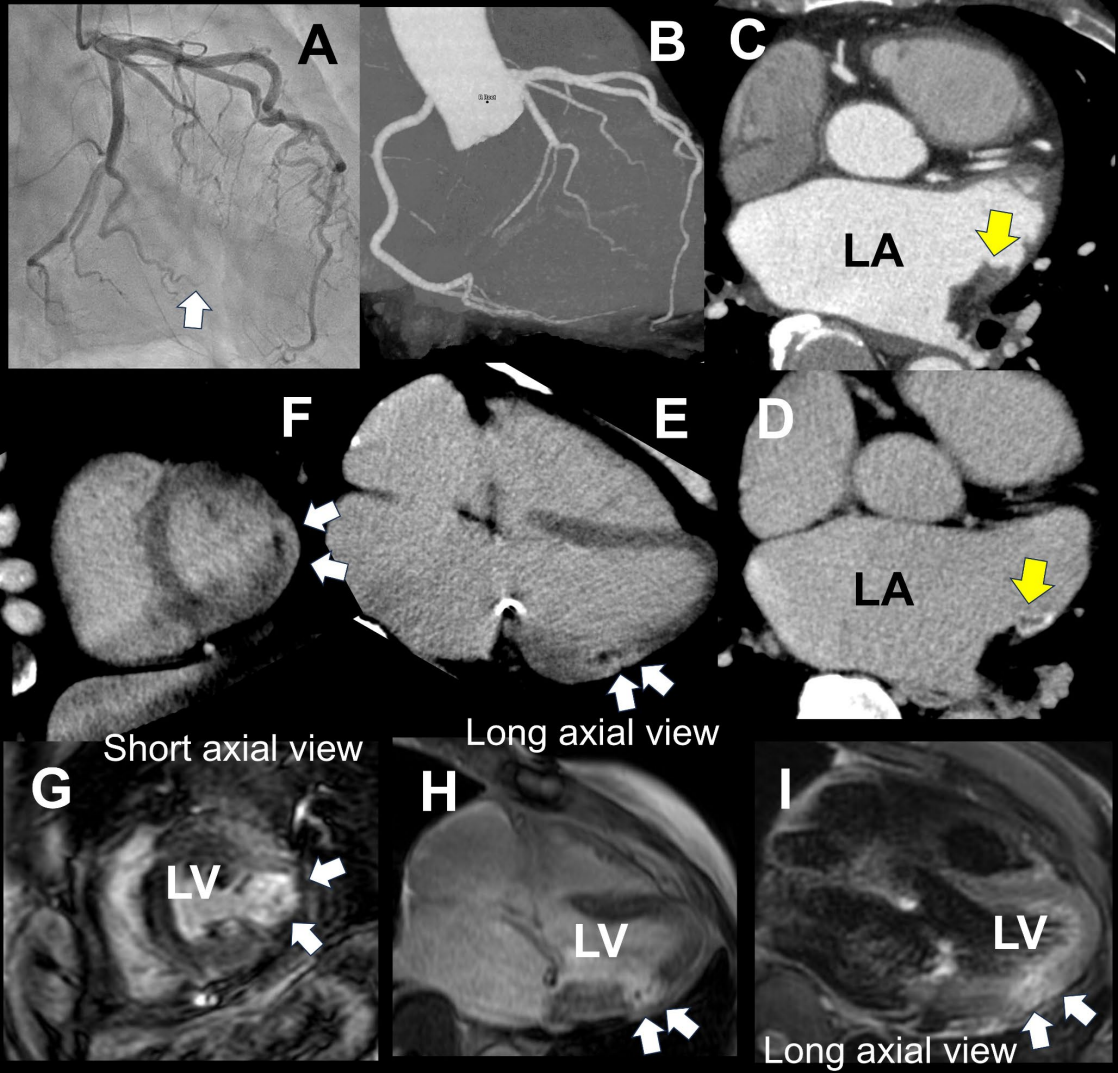

## Data Availability

No datasets were generated or analysed during the current study.
